# Location of unaccessible implant surface areas during debridement in simulated peri-implantitis therapy

**DOI:** 10.1186/s12903-017-0428-8

**Published:** 2017-11-28

**Authors:** Valerie Steiger-Ronay, Andrea Merlini, Daniel B. Wiedemeier, Patrick R. Schmidlin, Thomas Attin, Philipp Sahrmann

**Affiliations:** 1 Clinic of Preventive Dentistry, Periodontology and Cariology, Center of Dental Medicine, University of Zurich, Plattenstrasse 11, 8032 Zurich, Switzerland; 2 Clinic of Masticatory Disorders, Removable Prosthodontics, Geriatric and Special Care Dentistry, Center of Dental Medicine, University of Zurich, Zurich, Switzerland; 3 Statistical Services, Center of Dental Medicine, University of Zurich, Zurich, Switzerland

**Keywords:** Peri-implantitis, Debridement, Non-surgical, Surgical, Cleaning, Treatment, Gracey curette, Ultrasonic scaler, Air powder abrasive device, Implant, Thread, Surface

## Abstract

**Background:**

An in vitro model for peri-implantitis treatment was used to identify areas that are clinically difficult to clean by analyzing the pattern of residual stain after debridement with commonly employed instruments.

**Methods:**

Original data from two previous publications, which simulated surgical (SA) and non-surgical (NSA) implant debridement on two different implant systems respectively, were reanalyzed regarding the localization pattern of residual stains after instrumentation. Two blinded examiners evaluated standardized photographs of 360 initially ink-stained dental implants, which were cleaned at variable defect angulations (30, 60, or 90°), using different instrument types (Gracey curette, ultrasonic scaler or air powder abrasive device) and treatment approaches (SA or NSA). Predefined implant surface areas were graded for residual stain using scores ranging from one (stain-covered) to six (clean). Score differences between respective implant areas were tested for significance by pairwise comparisons using Wilcoxon-rank-sum-tests with a significance level α = 5%.

**Results:**

Best scores were found at the machined surface areas (SA: 5.58 ± 0.43, NSA: 4.76 ± 1.09), followed by the tips of the threads (SA: 4.29 ± 0.44, NSA: 4.43 ± 0.61), and areas between threads (SA: 3.79 ± 0.89, NSA: 2.42 ± 1.11). Apically facing threads were most difficult to clean (SA: 1.70 ± 0.92, NSA: 2.42 ± 1.11). Here, air powder abrasives provided the best results.

**Conclusion:**

Machined surfaces at the implant shoulder were well accessible and showed least amounts of residual stain. Apically facing thread surfaces constituted the area with most residual stain regardless of treatment approach.

## Background

The use of dental implants to replace missing teeth is considered to be a successful standard therapy with well-documented long-term survival rates [1–3]. Biological and technical complications, however, are a consequential and clinically relevant side effect of a rising number of implants being placed. Among these, the most common biological complication is peri-implantitis, an inflammatory reaction associated with loss of supporting bone around an implant in function [4]. With a reported prevalence of around 20% of patients and 10% of implants [5, 6] peri-implantitis presents the major risk factor for the long-term success of dental implant treatment.

Like in periodontal disease, the peri-implantitis-associated inflammatory reaction and tissue destruction occurs as a host response to biofilm residing on the implant surface [7]. Therefore all cause-related therapeutic approaches are mainly directed towards an effective mechanical biofilm removal [8, 9]. The success rate of peri-implantitis therapy, however, is still modest for both approaches. Non-surgical techniques are actually considered not to be predictively successful in cases of advanced peri-implantitis [10]. The surgical approach generally shows a wide range even for short-time success rates [11]. Apart from patient related factors, such as oral hygiene, history of periodontitis or smoking [12–14], especially local factors are important for the therapeutic outcome. Poor implant surface accessibility, further complicated by three-dimensional microstructures, thread design, pronounced taper in the implant shoulder area and platform switching make a complete removal of the biofilm almost impossible. Since biofilm is the primary etiologic factor for tissue inflammation, this should be highlighted as a crucial shortcoming of any peri-implantitis therapy. In this context, defect anatomy also plays an important role. For instance the presence of narrow vertical defects will result in inferior access and therefore cleanliness of the affected implant surface as compared to wide horizontal defects [15].

Two recent in vitro studies investigated the cleaning efficacy of common instruments typically applied during surgical and non-surgical implant debridement [16, 17]. The results of these investigations showed that regardless of the instrumentation technique used, the overall percentage of residual uncleaned surface was remarkably high. Defect configuration as well as the specific instrument applied did play an important role for the cleaning efficacy. However, any analysis of which exact implant areas are specifically difficult to clean is still missing. Knowledge regarding these non-accessed areas is the key for the development of more efficient instruments or techniques, which are aiming for the complete removal of pathogenic biofilm as the main culprit in the development und sustaining of peri-implantitis.

Therefore the aim of the present study was an analysis of the localization of residual stains in order to determine implant aspects, which are clinically challenging to clean during surgical and non-surgical instrumentation, based on the critical re-evaluation of two existing data sets. We hypothesized that apically facing thread surfaces constitute the most challenging areas presenting with most residual stain regardless of the treatment approach.

## Methods

Details on the data collection can be found in the original publications, which examined the surgical (SA) and nonsurgical (NSA) implant debridement approaches on two different implant systems [16, 17]. All analyses for the present study were performed on photographs previously taken in the course of the respective studies.

### Instrumentation

Two operators with different experience levels had performed all instrumentations. Treatment time was restricted to 120 s per implant. Three different instruments were used for the cleaning of the implant surface as follows:

1. A Gracey steel curette Nr. 11/12 (Deppeler, Rolle, Switzerland): standard use with the ring finger continuously applied as fulcrum.
2. An ultrasonic device with a steel tip (PiezoLED Scaler Tip 201, KaVo, Biberbach/Riss, Germany): use in gentle pressure-less movements in vertical and horizontal directions at maximum settings for “water cooling” and “power”.
3. An air powder abrasive device (AIR-FLOW Master®; EMS, Nyon, Switzerland) with glycine powder (AIR-FLOW® powder perio; EMS, Nyon, Switzerland) at maximum settings for “lavage” and “power”. For the SA, the common hand piece for supragingival instrumentation was applied, whereas for the NSA a nozzle for subgingival use was used. The latter had been used only once for each implant and was discarded afterwards.

### Defect models, implants and evaluated implant surface areas

Custom-made standardized models were made from polymethacrylate resin (Paladur clear®; Kulzer, Hanau, Germany) with three different defect morphologies, i.e. opening angulations of 30°, 60°, and 90° (horizontal defect). Each subgroup (defect type, type of instrument and treatment approach) consisted of 20 implants, resulting in a total of 360 analyzed implants. Implants were coated with water-insoluble, non-covering ink stain (Staedler permanent Lumocolor, Nürnberg, Germany) to simulate an optically identifiable biofilm surrogate.

#### Surgical approach

**Fig. 1:**
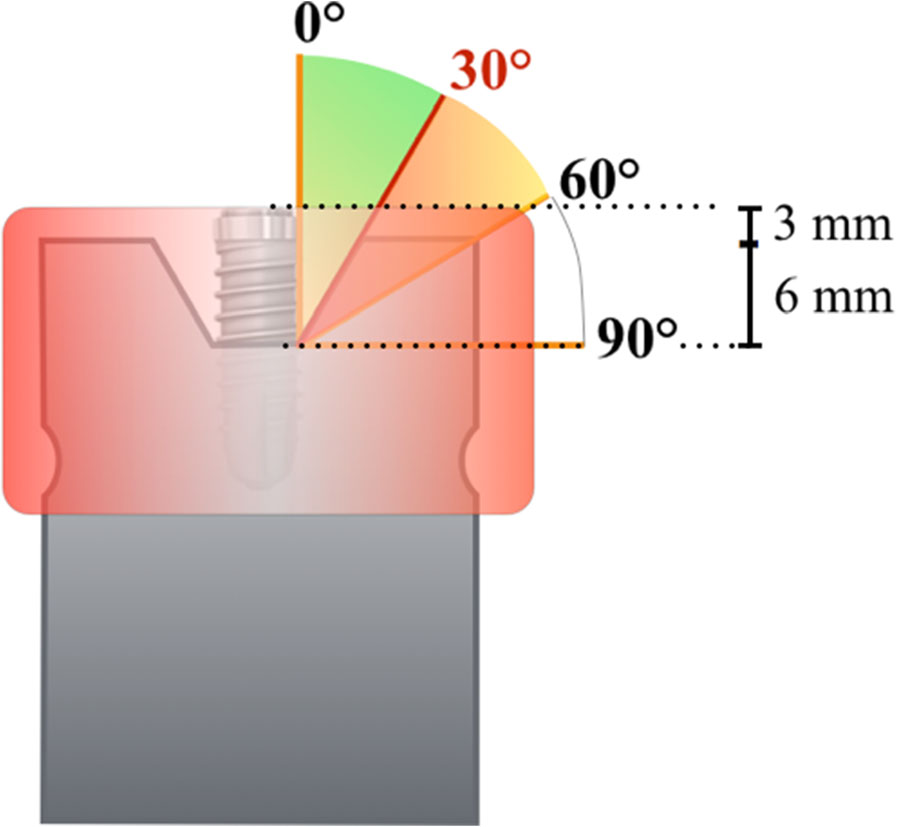
Schematic illustration of the custom-made standardized models with three different defect morphologies, i.e. opening angulations of 30°, 60°, and 90°. For the simulation of the non-surgical approach, an artificial mucosa mask (red) covered the mounted implant

To simulate the surgical approach, Straumann tapered effect wide neck implants (Straumann, Basel, Switzerland; length: 12 mm, diameter: 4.8 mm, shoulder diameter: 6.5 mm) with a micro-rough sandblasted and acid-etched (SLA) surface of 2-4 μm were used [16]. Implants were centrally mounted in the simulated defects in such a way that the rough surfaces leveled with the upper edge of the model for the imitation of circumferential peri-implant defects with a depth of 6 mm (see Fig. 1). Instrumentation was performed by a dental hygienist with over 35 years of clinical experience and a 2nd-year postgraduate student in periodontology. After instrumentation, implant surfaces were assessed for residual stain. Following predefined implant surface areas were assessed: tip of the threads with adjacent 0.5 mm (TT), apically facing thread surfaces from transition point to 0.5 mm before tip (AT), area between threads (BT), machined surface area (M) and rough shoulder area (R) at the implant shoulder.

#### Non-surgical approach

For the imitation of the non-surgical approach, SPI Element RC Inicell® implants (Thommen Medical, Grenchen, Switzerland, length: 11 mm, endosseous diameter: 4.2 mm) with a mean roughness of the endosseous surface of 2.35 ± 0.25 μm were used [17]. Implants were mounted in the same way as described above, butwere additionally covered by a non-transparent custom-made mucosa mask (opaque gelatine; gelatine ballistic type 1, Gelita, Eberbach, Germany). This mucosa mask had two functions: first it should prevent visual control of the performed cleaning. Second, it should make access to the stained implant surface more difficult. An experienced board-certified periodontist and a dental school graduate with less than 100 h of clinical training performed the implant instrumentation.

**Fig. 2:**
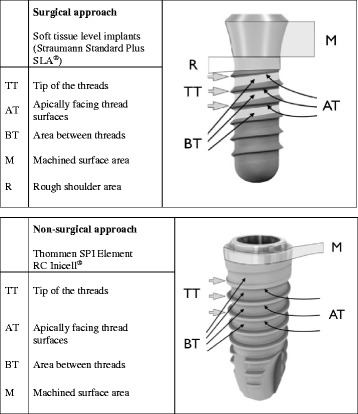
Surface areas analyzed in different approaches and implant types

Predefinition of implant surface areas was similar to the surgical approach: tip of the threads with adjacent 0.5 mm (TT), apically facing thread surfaces from transition point to 0.5 mm before tip (AT), area between threads (BT) and machined surface area (M).Since implant anatomy for the NSA was different at the marginal aspect, no R area was defined (see Fig. 2).

### Assessment of surface cleanliness

Digital photographs of the implant surfaces were taken with standardized parameters as described in more detail previously [16]. After instrumentation, implants were removed from the bases. Loosened ink particles were removed by gentle rinsing with water and air. Digital color photos were taken vertically to the implant axis with standardized parameters (dark chamber, ISO 100, aperture f/32, shutter speed 1/250 s, distance 31.4 cm with a Nikon D200, Tokyo, Japan, ring flash EM-140 DG; Metz MB 15 MS-1 Makroslave digital flash, Zirndorf, Germany, with power settings 1/2) from one side and the opposite aspect (180° turn). Ink remnants on the surface were detected using an image processing software (Adobe Photoshop Elements Vs. 9.0.3, Adobe Systems Inc., San Jose, CA, USA). The cumulative remnant area per implant was calculated using a custom-programmed planimetrical software (PPK, Zurich, Switzerland) [16]. Two blinded examiners independently assessed these photographs by grading the predefined implant surface areas for residual stain with a score ranging from one to six as follows: Score 1 indicated a surface almost completely covered by residual stain (>95%), score 2 a surface that was covered for more than ¾ (75–95%), score 3 for more than a half (50–75%), score 4 less than a half (25–50%), score 5 less than 25% clean but considerable residual stains (5–25%) and score 6 nearly perfectly clean surfaces (<5%). In cases of a divergence exceeding one score between the examiners, classification was reassessed by discussion.

### Statistics

Scores from the two examiners were analyzed for concordance using intra-class correlation coefficients (two-way model). Means, standard deviations as well as medians and interquartile ranges were calculated for the descriptive statistics of residual stain. Pairwise cleaning differences between implant surface areas were tested using Wilcoxon-rank-sum-tests. Resulting *p*-values were adjusted for multiple comparisons according to Holm and the significance level was set to α = 5%. Statistical analysis was performed in R [18], including the R package “irr” [19].

## Results

Irrespective of approach, when a score divergences exceeded 1 score, this difference was caused by an obvious classification mistake and was corrected by discussion.

### Surgical approach

**Fig. 3:**
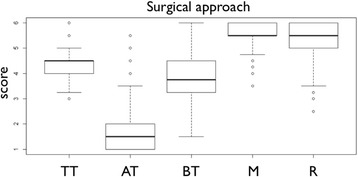
Boxplots presenting the scores for residual stain of the surgical approach. Medians with interquartile ranges are presented by boxplots. Scores were ranging from one (residual stain >95%) to six (residual stain <5%). TT - Tip of the threads, AT - Apically facing thread surfaces, BT - Area between threads, M - Machined surface area, R - Rough shoulder area

Intra-class correlation for the inter-rater reliability was determined to be 0.91. Residual stain scores are presented in Table 1a. Regardless of the instrumentation technique applied, differences in terms of stain removal were found between each of the analyzed areas (Fig. 3). Least residual stain was found for the machined surface area at the implants’ shoulder (5.58 ± 0.43), followed by the tips of the threads (4.29 ± 0.44), and the areas between the threads (3.79 ± 0.89). Apically facing thread surfaces constituted the area that presented with most residual stain (1.70 ± 0.92). When analyzing the residual stain ratings of apically facing threads in detail, cleaning efficacy was very low regardless of the applied instrument and analyzed defect type (Fig. 5a and Table 2a).

**Table 1:** Residual stain scores of the surgical (a) and non-surgical approach (b)

	TT	AT	BT	M	R
a) Surgical approach	4.29 ± 0.44 A	1.70 ± 0.92 B	3.79 ± 0.89 C	5.58 ± 0.43 D	5.41 ± 0.67 E
	4.50 (0.50)	1.50 (1.00)	3.75 (1.25)	5.50 (0.50)	5.50 (1.00)
b) Non-surgical approach	4.43 ± 0.61 A	1.65 ± 0.74 B	2.42 ± 1.11 C	4.76 ± 1.09 D	
	4.44 (0.50)	1.50 (1.00)	2.19 (2.00)	5.00 (2.00)	

Means ± standard deviations and medians (interquartile ranges), the latter in the second lines

Scores were ranging from 1 (residual stain >95%) to 6 (residual stain <5%)

*TT* Tip of the threads, *AT* Apically facing thread surfaces, *BT* Area between threads, *M* Machined surface area, *R* Rough shoulder area

Different capitals indicate groups with statistically significant differences (p < 0.001 for all comparisons except M vs. R where *p* = 0.01), as assessed by pairwise Wilcoxon-rank-sum-tests with p-value adjustments for multiple comparisons according to Holm

**Table 2:** Residual stain scores of the apically facing thread surfaces for the different instruments and the defects for the surgical approach (a) and non-surgical approach (b)

	30°	60°	90°
a) Surgical approach			
Gracey curette	1.31 ± 0.42 A	1.41 ± 0.45 A	1.32 ± 0.38 A
	1.00 (0.5)	1.50 (0.5)	1.25 (0.5)
Ultrasound	1.48 ± 0.41 A	1.34 ± 0.36 A	3.09 ± 1.07 B
	1.50 (0.5)	1.50 (0.5)	2.75 (1.5)
Air powder abrasion	1.25 ± 0.39 A	1.34 ± 0.49 A	2.79 ± 1.18 B
	1.00 (0.5)	1.00 (0.5)	2.75 (2.0)
b) Non-surgical approach			
Gracey curette	1.38 ± 0.16 A	1.3 ± 0.15 A	1.1 ± 0.15 CE
	1.5 (0.25)	1.25 (0.25)	1.0 (0.25)
Ultrasound	1.4 ± 0.17 A	1.09 ± 0.17 C	1.09 ± 0.12 CE
	1.5 (0.25)	1.0 (0.06)	1.0 (0.25)
Air powder abrasion	2.82 ± 0.47 B	1.96 ± 0.55 D	2.7 ± 0.7 BF
	2.75 (0.656)	1.81 (0.75)	2.5 (0.91)

Means ± standard deviations and medians (interquartile ranges), the latter as second lines in rows

Scores were ranging from one (residual stain >95%) to six (residual stain <5%)

Different capitals indicate groups with statistically significant differences (p < 0.001), as assessed by pairwise Wilcoxon-rank-sum-tests with p-value adjustments for multiple comparisons according to Holm

### Non-surgical approach

**Fig. 4:**
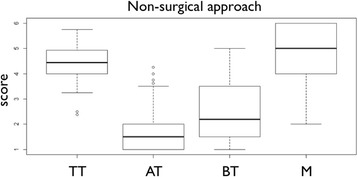
Boxplots presenting the scores for residual stain of the non-surgical approach. Medians with interquartile ranges are presented by boxplots. Scores were ranging from one (residual stain >95%) to six (residual stain <5%). TT - Tip of the threads, AT - Apically facing thread surfaces, BT - Area between threads, M - Machined surface area, R - Rough shoulder area

**Fig. 5:**
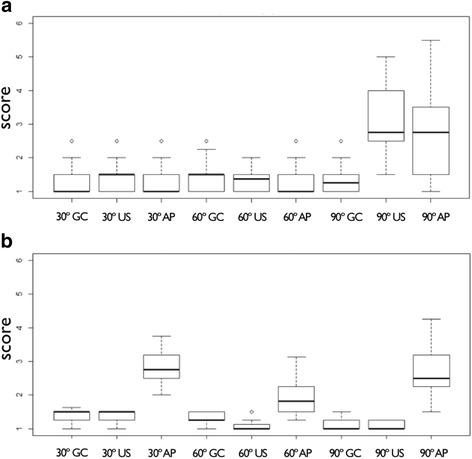
Boxplot presenting the scores for residual stain of the apically facing thread surfaces for the surgical (**a**) and the non-surgical (**b**) approach. Ratings are depicted for the different instruments (GC - Gracey curette, US - ultrasonic device, AP - air powder abrasive device) and defect angulations. Scores were ranging from one (residual stain >95%) to six (residual stain <5%)

Calculation of intra-class correlation for the inter-rater reliability resulted in 0.92. Table 1b presents the residual stain scores. Most residual stain was found at the apically facing thread surfaces (2.42 ± 1.11), while machined surface areas at the implants’ shoulder presented with least residual stain (4.76 ± 1.09). Differences in terms of stain removal were found between each of the analyzed areas, regardless of the instrument used (Fig. 4). Similar to the surgical approach, cleaning efficacy of apically facing threads was very low (Table 2b). However, with air powder abrasives cleaning was significantly better than for Gracey curettes and the ultrasonic device (*p* < 0.001) (Fig. 5b).

## Discussion

The aim of the present study was the detailed analysis of the localization of residual stain after implant instrumentation in order to identify implant areas, which are clinically difficult to access. In previous studies, we could already show that operator experience plays a significant role only when using Gracey curettes. However, since the observed differences were minimal and therefore clinically irrelevant, however, operator data was not itemized but pooled for the present examination.

Findings of both surgical and non-surgical approach were examined. Following main results were found: 1) Regardless of the applied cleaning approach and instrumentation type, significant differences in cleaning efficacy were found between the distinct implant surface areas. 2) Machined surface areas at the implant shoulder were generally well accessible and showed the least amounts of residual stain detectable. 3) Apically facing thread surfaces showed the most residual stain regardless of approach and instrumentation technique. Therefore the hypothesis, that apically facing thread surfaces constitute the most challenging areas was confirmed. In fact, these areas presented considerable amounts of residual stains. Although air powder abrasives provided the best cleaning results, still around ¾ of the surface remained uncleaned.

Pre-tests of the original studies showed, that coronally facing thread surfaces were almost perfectly cleaned [16, 17]. Therefore, photographs were focused on the more problematic apically facing threads surfaces to permit the semi-quantitative analysis of these difficult to clean areas.

For the area between the threads slightly better results were observed for the surgical approach than for the non-surgical therapy, even though a comparison is difficult because of the different implant systems investigated. This finding does not come as a surprise, since the aim of this intervention is namely better surface access and visual control. Differences between the two approaches in terms of scores obtained, however, were only moderate and not as pronounced as one might have expected. In part, this might be explainable by the use of different instruments in the SA and NSA: While glycine powder was applied with the conventional hand piece during the surgical approach, a special nozzle for subgingival (i.e. submucosal) use has been employed for the non-surgical approach. With this working tip, the powder flow is changed into a direction vertical to the instrument’s axis, which renders those areas potentially more accessible that can neither be controlled visually nor directly reached with a hand instrument. This could also be an explanation that for the NSA and air powder abrasives no significant cleaning difference was found between the 30° and 90° (horizontal) defect. It is possible that in the narrow 30° defect the subgingival nozzle was guided towards the implant surface which lead to results that are comparable with the horizontal defect.

Nowadays, peri-implantitis treatment is still unpredictable even in the short-term, as reflected in the recent literature [12, 20]. In this in vitro investigation, considerable amounts of residual stain remained for all modalities, even though surface debridement was performed under less complicated conditions i.e. without interference of the tongue, blood and saliva. This fact may strongly contribute to the reasons, why peri-implantitis treatment often fails. Noteworthy, it proves that we face innate limits that cannot be overcome with the equipment that is usually employed in modern peri-implantitis treatment concepts. Even with air power abrasives, which provided the best results in both investigations, only up to 25% of the apically facing thread surfaces were reached in the surgical approach. Accordingly, further efforts in instrument development should concentrate on these crucial surface areas.

An obvious limitation of the present study is the assessment of two different implant systems for the investigated approaches. This was done in order to not favor one specific implant system. Both implant types represent standard implants and depict classic designs with a machined implant neck, a micro-rough surface and comparable threads. Nevertheless, we decided to report the results for the SA and NSA and hereby the two implant types separately. Although they give a good indication as to which degree the surface of screw shaped implants may be generally debrided during SA and NSA, results must be compared with caution.

Another limitation is the fact that the present study is an in vitro investigation. The methods used simplified the clinical reality, but benefited from standardized, repeatable and easily assessable conditions. The use of ink stain instead of real biofilm needs to be discussed as well in this context, since different mechanical and adhesive properties are to be expected. The removal of indelible ink stain, however, is presumably more difficult than the disruption of actual biofilm. The optical detection of ink color remnants in photographs is reproducible and less fault prone than the assessment of biofilm, which is highly technique-sensitive and requires several analytical steps [21].

## Conclusion

Irrespective of the limitations of this in-vitro study apically facing threads seem not to be sufficiently cleanable. The development of new instruments must therefore focus on the effective debridement of these crucial areas in order to allow for reliable and predictable results in peri-implantitis treatment.

## Abbreviations

AT: Apically facing implant thread surfaces
BT: Area between implant threads
M: Machined implant surface area
NSA: Non-surgical implant debridement approach
R: Rough shoulder area at the implant shoulder
SA: Surgical implant debridement approach
TT: Tip of the implant threads
